# HFE hemochromatosis screening in patients with severe hip osteoarthritis: A prospective cross-sectional study

**DOI:** 10.1371/journal.pone.0207415

**Published:** 2018-11-14

**Authors:** Bastian Oppl, Emma Husar-Memmer, Svea Pfefferkorn, Martha Blank, Peter Zenz, Eva Gollob, Christian Wurnig, Alfred Engel, Andreas Stadlmayr, Gökhan Uyanik, Wolfgang Brozek, Klaus Klaushofer, Jochen Zwerina, Christian Datz

**Affiliations:** 1 Ludwig Boltzmann Institute of Osteology at the Hanusch Hospital of WGKK and AUVA Trauma Centre Meidling, 1st Medical Department, Hanusch Hospital, Vienna, Austria; 2 Orthopedic Centre, Otto-Wagner-Hospital, Karl Landsteiner Institute of Orthopedic Surgery, Vienna, Austria; 3 1st Orthopedic Department, Orthopedic Hospital Speising, Vienna, Austria; 4 Orthopedic Department, Sozialmedizinisches Zentrum Ost—Donau Hospital, Vienna, Austria; 5 Department of Internal Medicine, Hospital Oberndorf, Teaching Hospital of the Paracelsus Private Medical University of Salzburg, Oberndorf, Austria; 6 Centre for Medical Genetics, Hanusch Hospital and Faculty of Medicine, Sigmund Freud University, Vienna, Austria; Medizinische Fakultat der RWTH Aachen, GERMANY

## Abstract

**Objective:**

Despite the high frequency of *HFE* gene mutations in Western Europe, widespread screening for *HFE* hemochromatosis is not recommended due to its variable phenotype. Joint pain and a premature osteoarthritis-like disease including the hip joints are the most frequent manifestation in patients with *HFE* hemochromatosis and iron overload. Therefore, screening of patients with severe osteoarthritis of the hip could identify patients with *HFE* hemochromatosis.

**Methods:**

In this prospective cross-sectional study, 940 patients aged <70 years with end-stage osteoarthritis of the hip undergoing elective joint replacement surgery were screened for *HFE* hemochromatosis and compared to age- and sex-matched controls.

**Results:**

No greater prevalence of C282Y homozygosity mutation or elevated serum ferritin or transferrin saturation levels was found in the study cohort with severe osteoarthritis of the hip than in controls from the general population.

**Conclusion:**

Our screening approach could not identify an increased prevalence of *HFE* gene mutations and iron overload in younger patients with severe osteoarthritis of the hip.

## Introduction

*HFE* hemochromatosis (HH) is a systemic disorder leading to iron overload in parenchymatous organs, eventually causing organ failure. Clinically manifest HH is associated with high morbidity and increased mortality. The classical triad was characterized by liver cirrhosis, diabetes and skin hyperpigmentation. The majority of symptomatic patients harbor a homozygous mutation leading to a cysteine-to-tyrosine substitution at amino acid position 282 (C282Y) in the High Iron Fe (*HFE*) gene on chromosome 6, which leads to inappropiate increased iron absorption from the gut [1]. The homozygous prevalence of this autosomal-recessive mutation is ~0.5% in Western Europe [2]. Other mutations such as the H63D mutation (histidine to aspartic acid) of the *HFE* gene can also be found frequently, but are usually clinically not relevant [3].

Phenotypic expression of C282Y homozygosity is variable and depends on environmental and host factors [4] and polymorphisms of *TMPRSS6*, *GNPAT* and *PCSK7* genes relevant to iron absorption and cirrhosis risk [5, 6]. The rate of iron-overload-related disease in C282Y homozygotes was 28.4% for men and 1.2% for women in a large epidemiological study [7]. Joint pain often presents first and is the most common manifestation of HH. While symptoms do not differ from primary osteoarthritis (OA), the typical distribution of affected joints found in HH is remarkable. Metacarpophalangeal and ankle joints are frequently affected in HH, while hip joints are regularly involved in both disorders [8]. Site-specific arthropathy may be influenced by iron overload in HH patients. Arthropathy in HH was associated with iron overload in some studies [9], but not in others [10]. However, HH individuals seem to develop hip joint arthropathy earlier in life than patients with primary OA [11]. Therefore, we hypothesized that screening younger patients with severe OA of the hip could identify HH patients.

## Patients and methods

### Study population

Patients with severe OA of the hip were recruited sequentially in three orthopedic departments in Vienna over a period of three years for this cross-sectional study to identify clinically symptomatic HH patients. Individuals aged between 18 and 70 years old diagnosed with severe OA of the hip and scheduled for elective joint replacement surgery of the hip were eligible. Patients undergoing joint replacement surgery due to traumatic fractures and revision procedures were not included. Between 2012 and 2014 a total of 1001 patients gave written informed consent to participate in the study. After exclusion of patients with missing data, 940 patients could be included in the study. As control group, 940 age- and sex-matched individuals from a population-based prospective study without history of joint replacement surgery were randomly selected [12]. The study was approved by the Ethics Committee of the City of Vienna (EK 11-103-0811).

### Patient assessment

Epidemiological data including age, sex and body mass index as well as history of former joint replacement surgeries were assessed. Iron status with serum ferritin (μg/l) and transferrin saturation (%) levels was obtained after overnight fasting and measured using standard laboratory methods. Blood samples for *HFE* genotyping were stored until further processing.

### HFE gene mutation analysis

DNA was isolated from 300 μl of EDTA blood using Maxwell® 16 LEV Blood DNA Purification Kits (Promega) and the Maxwell® 16 IVD System (Promega) according to the manufacturer's protocol. DNA samples were analysed for C282Y and H63D mutations of the *HFE* gene by Sanger sequencing. Primer sets for PCR amplification of human *HFE* coding exons 2 and 4 were designed using Primer3Plus software. The resulting PCR products were subjected to fluorescence-based cycle sequencing using the BigDye® Terminator Cycle Sequencing Ready Reaction Kit, version 3.1 (Applied Biosystems). Samples were run and analysed on an Applied Biosystems® 3500 Genetic Analyzer. Sequencing electropherograms were assessed by visual inspection to identify C282Y and H63D mutations.

### Statistical analysis

A three-fold increase in C282Y homozygous mutations in our patient cohort compared to the general population was estimated [11]. Statistical power for Fisher's exact test assuming alpha of 0.05 (two-tailed) was calculated using G*Power software (University of Duesseldorf, Germany). A case number of 2000 had 80% power to detect the assumed differences between groups. After a pre-planned halftime analysis the study was terminated early because of unexpectedly low prevalence of C282Y homozygosity in the patient group.

Data are presented as mean ± standard deviation (SD). Ferritin and transferrin saturation levels of patients with severe OA of the hip and controls were compared using Mann-Whitney U test. Distribution of genotypes between groups was analyzed by Pearson's chi-square test. Multivariate logistic regression analysis was used to determine the association of HFE genotypes with severe hip OA, adjusting for covariates age, sex, body mass index, history of diabetes and serum ferritin level. P-values <0.05 were considered statistically significant. SPSS statistical software (SPSS Inc., Chicago, IL) was used for statistical analysis.

## Results

Mean age of 940 patients undergoing hip replacement surgery was 59.4 ± 9.5 years and ranged from 19 to 70 years. Male to female ratio of patients enrolled in the study was 1:1.4 and women were 1.7 years older in average. With a mean BMI of 27.9 ± 5.1, in overall patients could be classified pre-obese according to the World Health Organization. Mean BMI in the control group was 27.0 ± 4.8 (Table 1).

**Table 1:** Iron status and *HFE* genotype in patients with severe ostearthritis of the hip (OA) and controls.

	OA, n = 940	Control, n = 940	p		
Men : women	385 : 555	385 : 555			
Age, years	59.4 ± 9.5	60.0 ± 10.1	0.27		
BMI	27.9 ± 5.1	27.0 ± 4.8	<0.05		
Ferritin, μg/l	151.4 ± 157.6	200.4 ± 329.2	<0.05		
Transferrin saturation, %	26.1 ± 12.0	27.8 ± 12.0	<0.05		
Elevated ferritin and transferrin saturation*	20	29			
N/A	57	108			
*HFE genotype*, *n (%)*			<0.05		
Wild type	650 (69.1)	624 (67.2)	0.36		
H63D/-	203 (21.6)	182 (19.6)	0.28		
H63D/H63D	13 (1.4)	13 (1.4)	0.98		
C282Y/-	63 (6.7)	82 (8.8)	0.09		
C282Y/C282Y	1 (0.1)	6 (0.6)	0.06	<0.05	
C282Y/H63D	10 (1.1)	22 (2.4)	<0.05		
N/A	0	11			

*Hyperferritinemia (≥150 μg/l in women, ≥300 μg/l in men) and increased transferrin saturation level (≥45%) according to National Institute of Health reference values.

**Fig 1:**
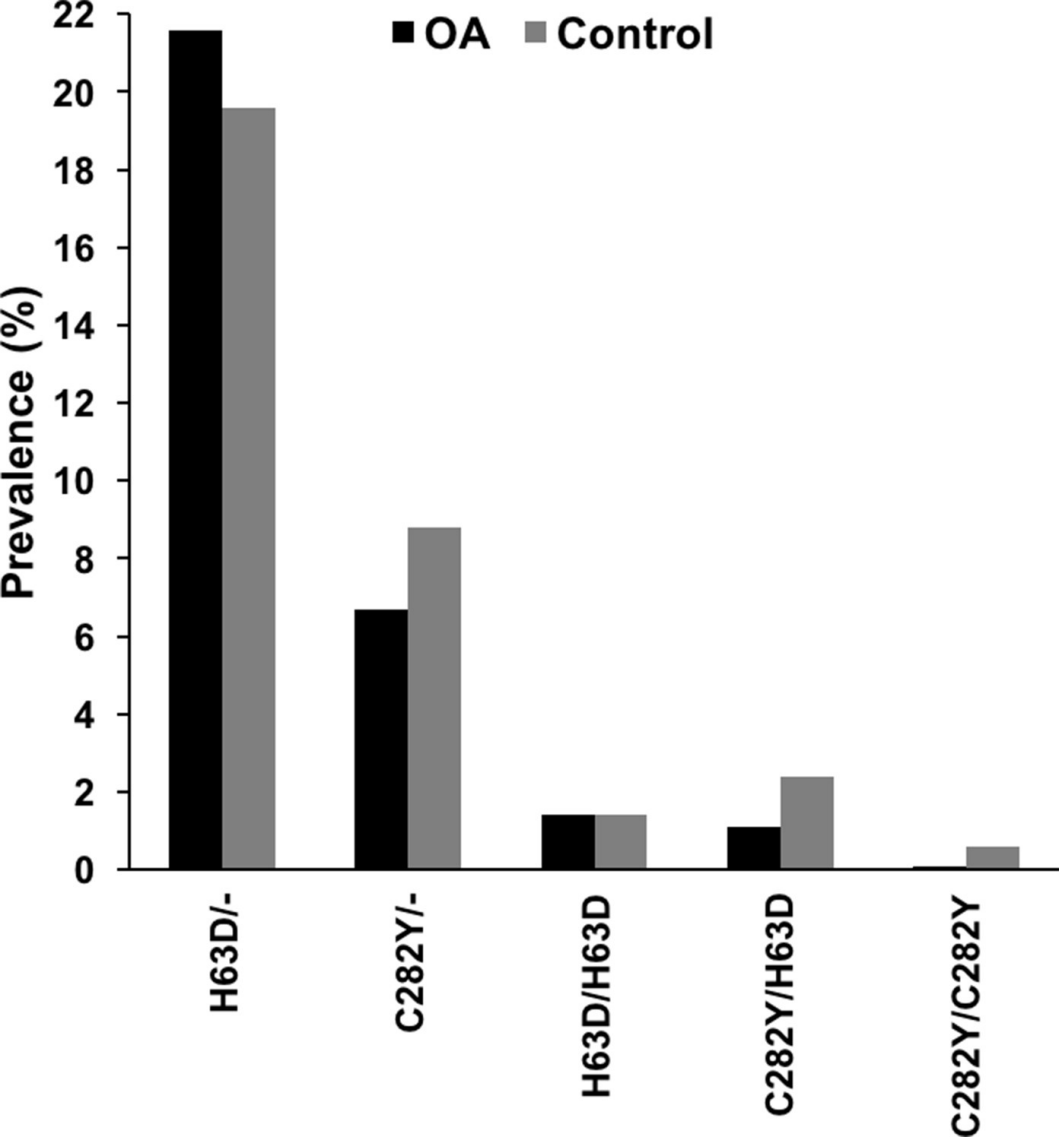
Prevalence of *HFE* genotype in patients with severe osteoarthritis of the hip (OA) and controls.

Serum ferritin and transferrin saturation levels were significantly higher in the control than in the patient group. C282Y homozygosity was found in only one (0.1%) out of 940 patients with end-stage OA of the hip. In the control group, six (0.6%) cases of C282Y homozygosity were identified. Compound heterozygosity (C282Y/H63D) was found in 10 (1.1%) versus 22 (2.4) of 940 individuals in the patient versus control cohort, respectively. Thus, C282Y homozygous and compound heterozygous mutations were significantly less prevalent in the hip OA group (p<0.05). According to this, multivariate regression analyses revealed a significantly lower prevalence of compound heterozygosity (C282Y/H63D) in OA patients than in controls (OR 0.44, 95%-CI 0.20–0.97, p<0.05). Prevalence of heterozygous C282Y and H63D mutations was 63 (6.7%) versus 82 (8.8%) and 203 (21.6%) versus 182 (19.6%) in the patient and control population, respectively. An equal number of 13 (1.4%) homozygous H63D mutations were found in both groups (Table 1 and Fig 1). *HFE* genotype was not available for 11 of 940 controls.

## Discussion

Tissue iron accumulation in HH can lead to major complications such as liver cirrhosis and hepatocellular carcinoma. Iron depletion by phlebotomy is the treatment of choice with potential reversal of hepatic fibrosis [13]. Thus, early diagnosis of the disease to prevent complications is important and screening of at-risk populations warranted. In this regard, a case finding approach by targeted screening of vulnerable individuals with signs suggestive of iron overload, as well as offering testing to Caucasian men of Northern European ancestry, has been discussed [14].

Joint pain is the most frequent presentation in patients with HH and iron overload and often precedes other symptoms [8]. In contrast to primary OA, HH arthropathy frequently affects men and shows symmetric joint involvement. Symptoms of the typically affected second and third metacarpophalangeal joints may be subtle and do not necessarily lead patients to consult rheumatologists. Bilateral ankle joint involvement is also typical in HH, but its prevalence in patients is quite low [15]. Other affected joint regions include the wrists, as well as knee and hip joints. Importantly, HH was established as a risk factor for hip replacement at younger age [11]. An increased risk of musculoskeletal complications was confirmed in two independent population-based studies in Sweden and Australia [16, 17].

As an approach for screening of at-risk populations, we analyzed serum iron status and *HFE* genotypes in patients with end-stage hip OA under the age of 70. Given the previous findings from epidemiological studies, we estimated a three-fold increase in C282Y homozygous mutations in our patient cohort compared to the general population. Nevertheless, our data did not reveal a significant increase in *HFE* mutations nor laboratory signs of iron overload in our study population compared to age- and sex-matched individuals. In fact, C282Y homozygosity was even lower in prevalence than in the control population, the latter being in the expected range. Thus, our study does not suggest a benefit from *HFE* screening in younger patients with severe OA of the hip. A possible reason for not detecting an increase in C282Y homozygotes in the OA group is that subjects in our study were not primarily selected by iron overload and few suffered from elevated serum ferritin and transferrin saturation levels. Further, it cannot be excluded that hemochromatosis patients with joint pain are being diagnosed and treated at earlier stages in Austria. In fact, many rheumatologists include full iron status in their diagnostic work-up of newly presenting patients. Also, a referral bias of certain patient groups cannot be fully excluded, although all recruiting centers are part of the social insurance system.

Hence, other case identification approaches have to be developed and tested in HH. Regarding hemochromatosis arthropathy, although rare, patients with severe ankle OA without mechanical trigger factors may serve as potential candidates. Although we could not prove a benefit in testing patients with severe hip OA for HH, studies for screening of at-risk populations need to be continued in order to prevent the course of this potentially life-limiting disease by its early detection and therapy.
